# Forage preservation (grazing vs. hay) fed to ewes affects the fatty acid profile of milk and *CPT1B* gene expression in the sheep mammary gland

**DOI:** 10.1186/1746-6148-8-106

**Published:** 2012-07-09

**Authors:** Elda Dervishi, Margalida Joy, Albina Sanz, Javier Alvarez-Rodriguez, Francisco Molino, Jorge H Calvo

**Affiliations:** 1 Unidad de Tecnología en Producción Animal, CITA, Zaragoza, Spain; 2 Departament de Producció Animal, Universitat de Lleida, Lleida, Spain; 3 Fundación ARAID, Zaragoza, Spain

## Abstract

**Background:**

Alterations in lipid metabolism occur when animals are exposed to different feeding systems. In the last few decades, the characterisation of genes involved in fat metabolism and technological advances have enabled the study of the effect of diet on the milk fatty acid (FA) profile in the mammary gland and aided in the elucidation of the mechanisms of the response to diet. The aim of this study was to evaluate the effect of different forage diets (grazing vs. hay) near the time of ewe parturition on the relationship between the fatty acid profile and gene expression in the mammary gland of the Churra Tensina sheep breed.

**Results:**

In this study, the forage type affected the C18:2 cis-9 trans-11 (CLA) and long-chain saturated fatty acid (LCFA) content, with higher percentages during grazing than during hay feeding. This may suggest that these FAs act as regulatory factors for the transcriptional control of the *carnitine palmitoyltransferase 1B* (*CPT1B)* gene, which was more highly expressed in the grazing group (GRE). The most highly expressed gene in the mammary gland at the fifth week of lactation is *CAAT/ enhancer- binding protein beta* (*CEBPB),* possibly due to its role in milk fat synthesis in the mammary gland. More stable housekeeping genes in the ovine mammary gland that would be appropriate for use in gene expression studies were *ribosomal protein L19* (*RPL19)* and *glyceraldehyde- 3- phosphate dehydrogenase* (*GAPDH).*

**Conclusions:**

Small changes in diet, such as the forage preservation (grazing vs. hay), can affect the milk fatty acid profile and the expression of the *CPT1B* gene, which is associated with the oxidation of fatty acids. When compared to hay fed indoors, grazing fresh low mountain pastures stimulates the milk content of CLA and LCFA via mammary uptake. In this sense, LCFA in milk may be acting as a regulatory factor for transcriptional control of the *CPT1B* gene, which was more highly expressed in the grazing group.

## Background

Sheep milk fat is rich in medium-chain triglycerides (MCT), which are composed of fatty acids with a carbon chain of 6–10 carbon atoms. MCTs are of special therapeutic interest because of their specific metabolism and their consequent application in certain types of metabolic illnesses [1]. By contrast, medium-chain saturated fatty acids, mainly C12:0 and C14:0, are considered to have a negative effect on human health when consumed in excess [2]. Other fatty acids (FAs), such as oleic (C18:1 n-9), linolenic (C18:3 n-3), eicosapentaenoic acid (EPA), docosahexaenoic acid (DHA) and conjugated linoleic acid (CLA, C18:2 cis-9 trans-11), have positive effects on human health [3,4]. Milk fat composition is affected by factors such as: nutrition, lactation stage and genetics. With diet having a high and immediate impact on milk FA composition in cows and goats [5,6], as well as in sheep [7-10].

In the last few decades, the characterisation of genes involved in fat metabolism and technological advances have enabled the study of the effect of diet on the milk FA profile within the mammary gland and aided in the elucidation of the mechanisms of the response to diet [11,12]. Studies that have examined the effects of diet on gene expression in sheep and goats under the same dietary treatment are limited [13,14]. In most studies, the feedstuffs used in animal diets have differed to relate the effects of diet to gene expression or enzymatic activity. Accordingly, grazing versus concentrate-rich diets [15,16] or the use of high-, medium- and low-quality diets [17] have been compared. However, few studies have explored the role of forage preservation on milk or meat FA profiles and the expression of genes related to fat metabolism. The hay making process results in the loss of fatty acids which act as precursors for CLA synthesis in the rumen and mammary gland [18]. Total FA was reduced by over 50% during hay making with a greater loss of linolenic acid (C18:3 n-3) [19]. To further address these issues, we studied the effect of forage preservation (grazing vs. hay) fed to ewes on the milk fatty acid profile and the expression of 8 key genes implicated in fat metabolism within the mammary gland: *lipoprotein lipase* (*LPL*), *acetyl-CoA carboxylase α* (*ACACA*), *fatty acid synthase* (*FASN*), *fatty acid binding protein 3* (*FABP3*) and *4* (*FABP4*), *diglyceride acyltransferase 1* (*DGAT1*), *stearoyl-CoA desaturase* (*SCD*) and *carnitine palmitoyltransferase 1B* (*CPT1B)*[16]*.* Lipoprotein lipase (LPL) is a rate-limiting enzyme in the hydrolysis of triglycerides circulating in the form of chylomicrons and very low-density lipoproteins into free fatty acids (FFAs) and 2-monoacylglycerols. The resulting FFAs can be utilised in different tissues, such as the mammary gland. The enzyme acetyl-coenzyme A carboxylase *α (*ACC), which is encoded by the *acetyl-CoA carboxylase α* gene (*ACACA)*, catalyses the ATP-dependent carboxylation of acetyl-CoA to form malonyl-CoA, which is the substrate for the synthesis of palmitic acid and long-chain fatty acids (acyl-CoA > C22:0) by the enzyme fatty acid synthase (FAS). In the presence of NADPH, FAS, which is encoded by the *FASN* gene, catalyses the synthesis of long-chain saturated fatty acids from palmitate derived from acetyl-CoA and malonyl-CoA. Fatty acid binding proteins 3 and 4, which are encoded by the *FABP3 and FABP4* genes*,* respectively, supply long-chain fatty acids as an important energy source and have been assumed to be mammary gland- and adipose tissue-specific, respectively. Carnitine palmitoyltransferase I (M- CPT 1), which is encoded by the *CPT1B* gene, is part of the mitochondrial transport system and is a key enzyme in the control of long-chain fatty acid oxidation. Stearoyl-CoA desaturase (SCD) is the rate-limiting enzyme that converts palmitoyl- and stearoyl-CoA to palmitoleoyl- and oleoyl-CoA, respectively. In ruminants, SCD is encoded by the *SCD* gene and participates in the formation of conjugated fatty acid (CLA) from C18:1 trans-11 in animal tissues. The *DGAT1* gene encodes the enzyme acyl-CoA:diacylglycerol acyltransferase1, which plays a central role in the synthesis of triglycerides. It catalyses the reaction of diacylglycerol and fatty acyl-CoA to form triglycerides. Most of these key enzymes are subject to both acute and chronic control, and their gene expression is regulated by important transcription factors such as *sterol regulatory element binding protein* (*SREBP1)peroxisome proliferator – activated receptor gamma* (*PPARG)* and *alpha* (*PPARA)*, and *CAAT/ enhancer- binding protein beta (CEBPB),* also studied in this work. Furthermore, the stability of housekeeping genes within the mammary gland for use in gene expression studies was assessed.

## Methods

### Experimental site and sheep breed

The experiment was conducted at La Garcipollera Research Station, in the Pyrenees (north-eastern Spain, 4237 N, 030 W, 945 m above sea level, a.s.l.), during autumn 2008. The average annual rainfall is bimodally distributed with peaks in spring and autumn, with dry summers and some precipitation in the form of snow in autumn and winter. The mean temperature during September, October, November and December was 16.2, 11.6, 5.0 and 3.0°C, while the precipitation in these months was 31.8, 120.6, 94.0 and 59.2 mm, respectively.

The breed used in this study was the Churra Tensina sheep, an endangered local coarse-wooled hardy breed (approximately 7,000 head) belonging to the Churra group that is raised for lamb production in the mountain area of the southern Pyrenees. The main Churra product is meat lamb, which can be of two commercial categories, suckling (10–12 kg LW) or light lamb (22–24 kg LW).

### Animal management and experimental design

Twenty-four multiparous single-bearing Churra Tensina ewes were used (body weight (BW) of 46.5 ± 0.66 kg and a body condition score (BCS) of 3.0 ± 0.03, on a 1 to 5 scale [20], at breeding). The flock was permanently grazing on medium-ranged mountain pastures (920–1500 m a.s.l.) during spring (68-72% neutral detergent fibre, NDF; 10-14% crude protein, CP). In summer (early to mid-pregnancy), the flock grazed on high mountain pastures (1500–2200 m a.s.l.) (68-75% NDF, 7-10% CP). The mating period was in spring from 5 May to 15 June, and the lambing was registered, on average, on 19 October ± 8 days. Four Churra Tensina rams were used for mating with the ewes.

To evaluate the effect of the diet (grazing vs. hay) on the relationship between the fatty acid profile and gene expression within the mammary gland of the Churra Tensina sheep breed, the experimental study began when pregnant ewes were in the last third of pregnancy (last 5 weeks) and finished at 5 weeks of lactation, when lambs reached the target BW of 10–12 kg for inclusion in the suckling lamb commercial category. Therefore, 5 weeks prior to parturition, ewes were divided into two groups:

· GRE treatment: 12 ewes were fed on mountain pastures *ad libitum* (43–46% NDF and 18–20% CP). During the pre-partum period, snow limited pasture availability for 11 days (15^th^-19^th^ and 22 ^th^ -27^th^ October), and ewes were offered 1 kg/day/ewe of alfalfa (*Medicago sativa*) pellets (35–37% NDF, 14–17% CP).

· HAY treatment: 12 ewes were fed pasture hay *ad libitum* indoors (49–52% NDF, 11–13% CP). This hay was made in late spring from the same pasture paddocks used by those grazed in autumn.

The pasture was composed of 22% legumes (mainly *Trifolium repens*), 68% grass (the main species were *Festuca arundinaceaFestuca pratensis* and *Dactylis glomerata*) and 10% other species (mainly *Rumex acetosa* and *Ranunculus bulbosus*) [21]. Outdoor grazing ewes had free access to a sheltered area.

### Measurements

The BW of ewes and lambs was recorded at weekly intervals at 8 a.m. with an electronic balance (0.1-kg precision). The BCS was measured according to Russel et al. [20] by two trained technicians at lambing and 5 weeks post-partum (drying off).

After lambing, milk production was recorded weekly by the oxytocin technique proposed by Doney et al. [22] with machine milking with hand finishing (4 h interval). Ewes were injected with 5 IU oxytocin in the jugular vein prior to machine milking and hand finishing at 08:00 and 12:00 h. Ewes were returned to their paddock between the two milkings, while lambs were confined. The milk obtained in the second milking was weighed, and the yield was extrapolated to the daily period (daily production = milk obtained x 6). Individual milk samples (50 ml) were preserved by adding potassium dichromate and stored at −20°C until future milk fatty acid analysis during the subsequent 3 months. The sample that was taken on the last day of lactation was used to study the relationship between milk FA composition and gene expression in the mammary gland.

### Mammary gland biopsy

At the fifth week of lactation, mammary gland tissue was collected with a biopsy needle (Tru-Cut®, CareFusion, France). The animal handling and procedures were in accordance with current European legislation (directive 86/609/EEC) [23] and supervised by the Animal Welfare Committee of the institution (protocol number 2009-01_MJT). Ewes were positioned on a lateral recumbence. The biopsy site of the mammary gland was prepared by cleaning with 10% Povidone-iodine solution (Lainco, S.A. Barcelona, Spain), and local anaesthesia was administered by subcutaneous injection of 2.5 ml lidocaine chlorhydrate (Xilocaina ovejero 2%, Ovejero Laboratorios, León, Spain). A 2-mm incision was made approximately 2 cm above the nipple to facilitate the insertion of the biopsy needle. The disposable needle had a specimen notch and a sheath over the needle to cut the tissue, which was necessary to withdraw the needle and sheath with the specimen from the mammary gland. After the biopsy, the mammary gland surface was sprayed with Aludemin (Chemical Ibérica, Salamanca, Spain) to protect the wound from dirt. Immediately after biopsy, the mammary tissue sample was stored in RNA later solution (QIAGEN, Madrid, Spain) and then frozen and stored at −80°C.

### Feed and milk fatty acid analysis

For the FA determination, feed samples were Soxhlet-extracted [24], and milk samples were submitted to a lipid separation as described by Luna et al. [25]. Both FA groups were converted to methyl esters by base-catalysed methanolysis of the glycerides with KOH according to the UNE-EN ISO 5509:2000 methods [26]. Fatty acid methyl esters were separated on a capillary column (HP-88 100 m x 0.25 mm ID and 0.20 μm film thickness, Agilent Technologies, Waldbronn, Germany) and detected with a flame ionisation detector (FID, gas chromatograph HP-6890). The carrier gas was helium, and the flow rate was 2 ml/min. The temperatures of the inlet and the detector were maintained at 250 and 300°C, respectively. The temperature program was as follows: the initial temperature was held at 100°C after injection, then programmed to increase at 1.5°C/min to 170°C (held there for 15 min), then programmed to increase at 0.5°C/min to 180°C (held there for 2 min), and finally to increase at 10°C/min to 215°C (held there for 20 min). The injection volume was 1.0 μl. Fatty acids were identified based on comparison with the retention times of a standard FA mixture (Sigma-Aldrich, Madrid, Spain). The individual fatty acid contents were expressed as weight percentages (g/100 g of total FA).

After determining individual FAs, the sum of saturated fatty acids (SFA), short-chain FA (SCFA; C4-C10), medium-chain FA (MCFA; C12-C14), long-chain FA (LCFA; C16-C24), total unsaturated FA (UFA), mono-unsaturated FA (MUFA) and poly-unsaturated FA (PUFA) were calculated, according to De la Fuente et al. [27]. Moreover, the UFA/SFA and n-6/n-3 ratios were determined.

### RNA extraction and cDNA synthesis

Total RNA was prepared from the mammary gland biopsy with TRI^@^REAGENT (Sigma-Aldrich, Madrid, Spain) according to the manufacturer's instructions. The concentration and quality of the RNA were determined by nanophotometric analysis (Implen, Madrid, Spain). To exclude possible amplification of contaminating genomic DNA, samples were treated with DNAse. Single-stranded cDNA was synthesised from 1 μg RNA with the SuperScript®III Reverse Transcriptase kit (Invitrogen, Carlsbad, CA, USA) following the manufacturer’s recommendations.

### Real-time polymerase chain reaction analysis (RT- PCR)

Gene expression levels were determined by RT-PCR on an ABI Prism 7500 platform (Applied Biosystems, Madrid, Spain). Primers for RT-PCR were designed with Primer3 software (http://frodo.wi.mit.edu/primer3/). The sequences of the primers and the RT-PCR conditions are described in Dervishi et al*.*[16]. In this study, the *FABP3* gene was also studied. The primer sequences were as follow: 5’- TTCAAGCTGGGAGTCGAGTT-3’ and 5’- TGTCCATTCCACTTCTGCAC-3’

RT-PCR was performed under the same conditions as the other genes with an annealing temperature, primer concentrations, correlation (R^2^) and slope of 60°C, [900, 900], 0.99 and −3.35, respectively. Before performing the RT-PCR reactions, a conventional PCR was performed for all genes to test the primers and verify the amplified products. The PCR products were sequenced to confirm gene identity with an ABI Prism3700 (Applied Biosystem, Madrid, Spain), and standard protocols. Homology searches were performed with BLAST (National Center for Biotechnology Information: http://www.ncbi.nlm.nih.gov/BLAST/) to confirm the identity of the amplified fragments. The PCR reaction was performed in a 10-μl PCR total reaction mixture containing SYBR Green PCR Master Mix (Applied Biosystem, Madrid, Spain). Each reaction was performed in triplicate, and the average was used to calculate the relative amount of the target gene. To normalise the results of the target genes, 8 candidate housekeeping genes were tested. In this study, the housekeeping genes analysed included those for ovine: *tyrosine 3-monooxygenase* (*YWHAZ*), *ribosomal protein L19*(*RPL19*), *glyceraldehyde-3-phosphate dehydrogenase* (*GAPDH*), *glucose-6-phosphate dehydrogenase* (*G6PDH*), *beta actin* (*ACTB*), *ubiquitin C* (*UBC*), *beta-2-microglobulin* (*B2M*), and *succinate dehydrogenase* (*SDHA*). The sequences of the primers and the RT-PCR conditions are described in Dervishi et al*.*[16]. Standard curves for genes were generated to calculate the amplification efficiency. The efficiency of PCR amplification for each gene was calculated with the standard curve method (E = 10^-1/slope^ -1). The standard curves for each gene were generated by 5-fold serial dilution of pooled cDNA. The amplification conditions were an initial step of 10 min at 95°C, followed by 40 cycles of 95°C for 15 sec and 59 or 60°C for 30 sec. The specificity of the amplification products and the lack of primer dimers were confirmed by melting curve analysis in all cases. To quantify the relative gene expression, the standard curve method was used according to the recommendation of Larionov et al. [28]. Normalised RT-PCR data were transformed to the fold-change relative to the control group, GRE; thus, data in the GRE group were transformed to obtain a perfect mean of 1. PCR-normalised data are presented as the *n*-fold relative change.

### Statistical analyses

Statistical analyses were performed with the SPSS statistical software package, version 19.0. To evaluate the effects of the forage type fed to the ewe on milk fatty acid content and gene expression in the mammary gland, the analyses were performed with a General Linear Model (GLM), in which the diet of the ewe was included as a fixed effect and the ewe body weight at lambing was included as a covariable, due to its possible relevance on the studied parameters during lactation [8]. The following equation was used for the model: Yijk = μ + Aj + b(Ek) + (A*b(E))jk + eijk, where Yijk = dependent variable; μ = overall mean; Aj = the effect of type of forage fed to the ewe; b(Ek) = body weight at lambing; b = linear regression coefficient; (A*b(E))jk = interaction between the forage type and body weight at lambing; eijk = residual error. Fatty acid results were expressed as least square means ± the standard error (SE) values, and the relative differences in gene expression between the groups were calculated and defined as the relative increase, setting the control means at 100%. To test the stability of housekeeping genes, we used *geNorm*[29] and *NormFinder*[30].

## Results and discussion

### Dietary composition

During the study period (70 days), pasture biomass decreased steadily from 14.4 ± 4.2 to 5.1 ± 0.6 cm. In the final pre-partum period, there was snow for 11 days during which 1 kg of alfalfa pellets was fed to each grazing ewe per day. Permanent pasture or alfalfa pellets were the dietary components of the grazing treatment (GRE), whereas the diet of the hay treatment (HAY) was pasture hay made in late spring from the same pasture paddocks used for autumn grazing (Table 1). The daily voluntary hay intake during the last 5 weeks of pregnancy was, on average, 1.38 ± 0.31 kg/ewe (as-fed basis), and this increased to 1.55 ± 0.31 kg/ewe (as-fed basis) during the 5 weeks of lactation.

**Table 1:** **Chemical composition (% of dry matter, DM) and fatty acid content (in % of total fatty acid methyl esters, FAME) of pasture hay, fresh pasture grazed and alfalfa pellets fed to Churra Tensina ewes during the peripartum period**^1^

**Diet**	**HAY**	**GRE**	
	**pasture hay**	**fresh pasture grazed**	**alfalfa pellets**^2^
***Chemical composition***			
Dry matter M (DM, % of fresh matter)	87.5	18.9	93.3
Crude protein, CP	12.0	20.1	15.7
Neutral detergent fibre, NDF	50.4	42.3	35.5
Acid detergent fibre, ADF	26.8	21.6	23.8
***Fatty acids***			
C12:0	0.38	0.23	0.66
C14:0	0.65	0.68	1.14
C16:0	14.13	15.54	13.94
C16:1 cis-9	0.40	0.77	0.21
C17:0	0.73	0.27	0.32
C18:0	3.35	3.02	2.80
C18:1 n-9	3.38	2.98	3.71
C18:1 trans-11 (VA)	0.11	0.20	0.02
C18:2 cis-9 cis-12	7.53	8.51	18.65
C18:3 n-6	1.94	0.54	0.30
C18:3 n-3	14	21.89	18.90
C20:0	18.82	23.44	23.01
SFA	38.60	43.19	37.76
MCFA	1.03	0.91	1.80
LCFA	36.30	42.00	39.75
UFA	27.36	34.89	45.90
MUFA	3.89	3.95	3.94
PUFA	23.47	30.94	41.96
PUFA n-6	9.47	9.05	18.95
PUFA n-3	14	21.89	23.01
n-6/n-3	0.68	0.41	0.82
UFA/SFA	0.71	0.81	1.22

^1^SFAs (saturated fatty acids) were calculated as the sum of C12:0 + C14:0 + C16:0 + C17:0 + C18:0 + C20:0; MCFAs (medium-chain fatty acids) were calculated as the sum of C12:0 + C14:0; LCFAs (long-chain fatty acids) were calculated as the sum of C16:0 + C18:0 + C20:0; UFAs (unsaturated fatty acids) were calculated as the sum of (MUFA + PUFA); MUFAs (monounsaturated fatty acids) were calculated as the sum of (C16:1 + C17:1 + C18:1n-9 + C18:11n-7 + C20:1); PUFAs (polyunsaturated fatty acids) were calculated as the sum of (C18:2 t-t + C18:2c-c + C18:3n-6 + C18:3n-3 + CLA + C20:2 + C20:3 + C20:4 + C20:5 + C22:5 + C22:6).

^2^Supplement during 11 days in the pre-partum grazing ewes, when snow limited pasture availability.

The dietary chemical composition and fatty acid profile are shown in Table 1. The estimated dietary metabolisable energy (ME) content according to Cannas et al. [31] was 9.1 and 10.2 MJ ME/kg DM for pasture and hay, respectively. In addition, the alfalfa pellets supplied 10.2 MJ ME/kg DM to grazing ewes when snow prevented pasture grazing. The higher CP and lower fibre (NDF and acid detergent fibre, ADF) contents in fresh pasture compared to hay indicate that grazed herbage had an early stage of growth and maintained vegetative characteristics during the experiment. Both feeding systems offered rather similar estimated energy contents, and crude protein was higher in GRE than in HAY.

The ewes BCS at lambing was 2.69 and 2.61 ± 0.07 in HAY and GRE ewes, respectively, while at drying-off, it was 2.32 and 2.45 ± 0.08, respectively. Although the forages supplied (fresh or hay) met the theoretical energy and protein requirements for milk production [32], there was BCS loss between lambing and drying-off in both treatments, suggesting that the voluntary intake of ewes did not meet the requirements for avoiding a negative energy balance.

The feedstuffs used in the GRE treatment (fresh pasture and alfalfa pellets) presented a 31.4% greater overall proportion of C18:3 n-3 than HAY feed. Alfalfa pellets exhibited a greater proportion of C18:2 cis-9 cis-12 and a lower proportion of C18:1 trans-11 (VA) than the other forages, but it is expected to exert only a minor influence due to the limited amount of intake. Hay feed had a 78.4% greater proportion of C18:3 n-6 than the other forages. By contrast, fresh pasture had a 36.05 and 16.66% greater proportion of C18:3 n-3 than hay feed and alfalfa pellets. These results are in agreement with Biondi et al. [33], who also observed that C18:3 n-3 was predominant in fresh pasture whereas C18:3 n-6 was more important in hay feed.

The sums of LCFA, total PUFA and PUFA n-3 were 11.2%, 35.6% and 37.6% greater in the feedstuffs used in the GRE treatment (fresh pasture and alfalfa pellets) than in the HAY treatment. Forage FA composition depends on the species and variety of the plant, the phenological phase of the plant and the preservation of the plant [34,35].

### Milk fatty acid profile

The fatty acid composition of milk at the fifth week of lactation is shown in Table 2. There were significant effects of forage type on C17:1 cis-10, C18:0, C18:2 cis-9 cis-12, CLA and C20:2 cis-11 cis-14 (P < 0.01) contents. The remaining milk FA was not significantly affected by forage type. Ewes belonging to the HAY group exhibited a higher content of C17:1 cis-10 and C18:2 cis-9 cis-12 than the GRE group. By contrast, the C18:0, CLA and C20:2 contents were higher in the GRE group (P < 0.01). The animal diets in both treatments were based exclusively on forage and the only difference was forage preservation, linked to plant phenological phase. Cabiddu et al. [36] also observed higher CLA and C18:3 n-3 contents in milk when ewes grazed pasture in the vegetative phase, whereas the CLA content decreased considerably when the same pasture was in the reproductive phase. In the present study, the most remarkable difference among forages was the greater C18:3 n-3 content in the fresh pasture compared to hay feed, in agreement with the loss of FA precursors of CLA caused by the hay-making process [10]. The lack of effect of diet on most of the milk FA composition contradicts reports in the literature [7,8,19,37], although most of these studies compared the effect of forage vs. concentrate diets.

**Table 2:** **Fatty acid content (in %) in milk of Churra Tensina ewes at the fifth week of lactation, according to forage type**^1–2^

**Fatty acid**	**Diet**			
	**HAY**	**GRE**	**SE**	***P-value***
C4:0	2.46	2.76	0.16	0.08
C6:0	1.77	1.92	0.09	0.14
C8:0	1.74	1.78	0.13	0.79
C10:0	5.08	4.62	0.53	0.39
C12:0	3.02	2.43	0.29	0.06
C14:0	7.87	6.70	0.62	0.07
C16:0	21.66	20.01	0.85	0.06
C16:1 cis-9	1.17	1.09	0.06	0.21
C17:0	1.27	1.32	0.07	0.45
C17:1 cis-10	0.70^a^	0.57^b^	0.04	0.01
C18:0	12.03^a^	14.60^b^	0.94	0.01
C18:1 n-9	27.67	28.56	1.66	0.59
C18:1 n-7	0.52	0.53	0.05	0.75
C18:2 trans-9 trans-12	0.24	0.28	0.02	0.12
C18:2 cis-9 cis-12	2.21^a^	1.88^b^	0.11	0.008
CLA C18:2 cis-9 trans-11	1.17^a^	1.58^b^	0.13	0.005
C18:3 n-6	0.037	0.004	0.003	0.84
C18:3 n-3	1.48	1.52	0.10	0.70
C20:0	0.20	0.18	0.01	0.18
C20:1 n-9	0.07	0.06	0.007	0.06
C20:2 cis-11 cis-14	0.10^a^	0.16^b^	0.02	0.004
C20:3 cis-8 cis-11 cis-14	0.002	0.002	0.003	0.94
C20:4 n-6	0.17	0.16	0.01	0.33
C20:5 n-3	0.10	0.10	0.01	0.92
C22:0	0.11	0.11	0.01	0.89
C22:5 n-3	0.20	0.19	0.01	0.77
C22:6 n-3	0.07	0.05	0.01	0.38
C24:0	0.053	0.040	0.01	0.27

^1^Within a row, means without a common superscript differ *(P < 0.05).* SE is the standard error.

^2^HAY group: ewes received hay from mountain pastures; GRE group: ewes grazed low mountain pastures.

Milk from grazing ewes contained higher levels of CLA than those fed hay indoors. Similarly, higher contents of CLA and vaccenic acid (VA, C18:1 trans-11) in milk and dairy products were observed when animals grazed fresh pastures compared to dried forages [38]. Grazing pasture may enhance the growth of specific bacteria in the rumen, stimulating the production of CLA and/or blocking the final reduction of VA to stearic acid (C18:0) [38], as well as increasing PUFA n-3 [5,34]. In the present study, grazing increased milk CLA content. In this sense, fresh pasture and alfalfa are richer in C18:3n-3 (21.89 and 18.90 vs. 14.00 for GRE and alfalfa vs. HAY, respectively), which can stimulate the CLA production.

Milk FAs were grouped according to the length and degree of saturation from a health perspective or according to ratios related to the human health (Table 3). Forage preservation affected only the LCFA and PUFA n-6 groups (P < 0.05). GRE ewes presented a significantly greater proportion of LCFA in milk than the HAY group, which agrees with their higher proportion in grazed pasture as well in dehydrated alfalfa compared to hay feed (Table 1). Total PUFA n-6 milk fatty acids were greater in the HAY group than in the GRE group due to the greater amount of linoleic acid (C18:2 cis-9 cis-12) in milk from HAY ewes than in milk from GRE ones. Collectively, the present study demonstrated that even small changes in diet, such as the forage preservation form, can affect the milk fatty acid profile.

**Table 3:** **Main FA categories content (%) in milk of Churra Tensina ewes at the fifth week of lactation, according to forage type**^1–3^

**Fatty acid**	**Diet**			
	**HAY**	**GRE**	**SE**	***P-value***
SFA	57.27	56.46	1.47	0.58
SCFA	11.06	11.07	0.68	0.98
MCFA	10.89	9.12	0.90	0.06
LCFA	35.32 ^a^	36.26 ^b^	0.40	0.03
UFA	36.80	35.90	1.70	0.61
MUFA	31.02	29.92	1.71	0.53
PUFA	5.96	5.75	0.21	0.34
UFA/SFA	0.65	0.64	0.04	0.75
PUFA n-6	2.65 ^a^	2.35 ^b^	0.12	0.02
PUFA n-3	1.84	1.88	0.12	0.83
n-6/n 3	1.49	1.28	0.12	0.10

^1^Within a row, means without a common superscript differ (P < 0.05). SE is the standard error.

^2^HAY group: ewes received hay from mountain pastures; GRE group: ewes grazed low mountain pastures.

^3^SFAs (saturated fatty acids) were calculated as the sum of C4:0 + C6:0 + C8:0 + C10:0 + C12:0 + C14:0 + C16:0 + C17:0 + C18:0 + C20:0 + C22:0 + C24:0; SCFAs (short-chain fatty acids) were calculated as the sum of C4:0 + C6:0 + C8:0 + C10:0; MCFAs (medium-chain fatty acids) were calculates as the sum of C12:0 + C14:0; LCFAs (long-chain fatty acids) were calculated as the sum of C16:0 + C18:0 + C20:0 + C22:0 + C24:0; UFAs (unsaturated fatty acids) were calculated as the sum of MUFA + PUFA; MUFAs (monounsaturated fatty acids) were calculated as the sum of C16:1 + C17:1 + C18:1n-9 + C18:11n-7 + C20:1; PUFAs (polyunsaturated fatty acids) were calculated as the sum of C18:2 t-t + C18:2c-c + C18:3n-6 + C18:3n-3 + CLA + C20:2 + C20:3 + C20:4 + C20:5 + C22:5 + C22:6.

### Selection of optimal housekeeping genes

To normalise the mammary gland gene expression results, we examined the stability of eight housekeeping genes with two different programs, *geNorm* and *NormFinder*. More stable genes analysed with the *geNorm* program were *GAPDH* (M = 0.60), *G6PDH* (M = 0.651) and *RPL19* (M = 0.825). When analysed with *NormFinder*, the *RPL19* (stability value = 0.11) and *GAPDH* (stability value = 0.11) genes still exhibited higher levels of gene expression stability. To normalise the data, we used the geometric mean of the *RPL19* and *GAPDH* genes. Both programs determined the most stable housekeeping genes, and we decided to use the most stable genes determined with the *NormFinder* program because this program considers the type of treatment, which was one of the most important factors.

### Real-time polymerase chain reaction analysis (RT-PCR) and the effect of feeding system on gene expression

**Figure 1:**
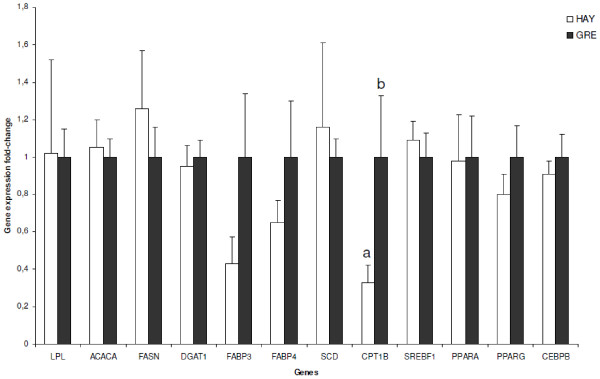
**Relative gene expressions in the mammary gland of the Churra Tensina sheep breed.** HAY group: ewes received hay from mountain pastures; GRE group: ewes grazed low mountain pastures. Different subscripts differ with at least P < 0.05.

In the present study, we analysed the expression of 12 genes related to lipid metabolism within the mammary gland. We found significant differences only in the expression of the *CPT1B* gene (Figure 1). We did not observe differences in the expression of the rest of genes, which may be due to the similarity of the diets. The *CPT1B* gene, which is associated with the oxidation of fatty acids, is expressed 0.34-fold lower in HAY ewes than in GRE ewes. The enzyme carnitine palmitoyltransferase (CPT1) is located in the outer mitochondrial membrane and represents the main site of control for the entry of LCFA into the mitochondria [39]. CPT1 catalyses the synthesis of long-chain acylcarnitines from long-chain CoA esters, and its activity has a high degree of control over the rate of fatty acid oxidation. Furthermore, in the liver, the utilisation of fatty acids for ketogenesis is also controlled by CPT1. CPT1 regulation of oxidation would suggest that the treatments altered energy balance more on the GRE ewes, which may be driven by exercise or lower intake. Furthermore, the promoter of *CPT1B* could be regulated by LCFA levels, and it has been previously reported that LCFAs are important regulatory factors for transcriptional control of *CPT1* in the rat neonatal liver [40] and in rat primary cardiac myocytes. Furthermore, a response element regulated by LCFA levels has been reported for both the rat and human *CPT1B* promoters [41-43]. In the present work, the forage type only affected the LCFA content of milk, demonstrating that LCFA mammary gland uptake was stimulated in grazing compared to hay-fed ewes. In this sense, LCFA may be acting as a regulatory factor for transcriptional control of the *CPT1B* gene. As this gene lacks TATA boxes, the baseline transcription regulation relies on transcription factor specificity protein-1 (Sp1) [44]. The induction of the *CPT1B* gene occurs via the fatty acid response element (FARE) or peroxisome proliferator-activated receptor (PPAR) response elements (PPREs), which bind the transcription factor PPAR [41,45]. However, in our study, we did not observe significant differences in the expression of either the *PPARA* or *PPARG* gene in the mammary gland at the fifth week of lactation. Therefore, LCFA may regulate *CPT1B* gene expression via another mechanism. M-CPT1 is subject to regulation at the transcriptional level and to acute control by malonyl-CoA [46]. The N-terminal domain of the M-CPT1 enzyme is essential for malonyl-CoA inhibition [47]. Rasmussen and Winder [48] suggested that muscle contraction inhibits acetyl-CoA carboxylase α (ACC) and would be one mechanism for increased fatty acid oxidation. A decrease in malonyl-CoA (during starvation) promotes fatty acid oxidation because M-CPT1 becomes uninhibited, whereas an increase in malonyl-CoA levels (during a fed state) will decrease mitochondrial fatty acid uptake and oxidation [49]. In the HAY group, ewes were fed pasture hay indoors *ad libitum*, while GRE group ewes were grazing fresh pasture throughout the experiment. In this sense, the treatments altered energy balance more on the GRE ewes, which may be driven by exercise. However, intake differences and other compounds such as protein and the plant secondary metabolites could also influence the results. Future studies are being planned to verify these factors not studied in the present work.

These results are in complete agreement with those obtained previously by Dervishi et al. [16], in which grazing systems promoted higher levels of CPT1B gene expression in the *semitendinous* muscle and fatty acid oxidation for energy production. These results suggest that grazing promotes higher levels of *CPT1B* gene expression in the mammary gland at the fifth week of lactation.

The results also revealed that *CEBPB* is the most highly expressed gene among all of the genes studied in the mammary gland at the fifth week of lactation (data not shown). Shi et al. [50] have also suggested a role for this factor in milk-fat synthesis. The activity of this factor may be a key regulator of the nutritional control of *ACACA* expression in lipogenic tissues. *SREBF1* was also highly expressed, and a central role for this gene as a mediator of FA effects has been outlined [51].

## Conclusion

In conclusion, low mountain pastures increased the milk content of C18:2 cis-9 trans-11 (CLA) and long chain fatty acids (LCFA) via mammary uptake, relative to hay fed indoors. *CPT1B* gene was more highly expressed in the grazing group possibly due to an altered energy balance by reducing energy intake and increasing exercise. Furthermore, LCFA in milk may be also acting as a regulatory factor for the transcriptional control of the *CPT1B* gene. More stable housekeeping genes in the ovine mammary gland for gene expression studies were *RPL19* and *GAPDH.* It was demonstrated that small changes in diet, such as forage preservation, can cause differences in the milk fatty acid profile and in the expression of *CPT1B.*

## Authors’ contributions

ED conducted the research and the statistical analysis. ED, MJ and JHC wrote the manuscript. AS and JAR contributed to the statistical analysis and the manuscript revision. FM performed the feed and milk fatty acid analysis. MJ and JHC designed the research. MJ provided animals. JHC has primary responsibility for the final content. All of the authors contributed to the manuscript discussion. All of the authors read and approved the final manuscript.
